# Comparison of ^68^Ga-FAPI and ^18^F-FDG PET/CT for the diagnosis of primary and metastatic lesions in abdominal and pelvic malignancies: A systematic review and meta-analysis

**DOI:** 10.3389/fonc.2023.1093861

**Published:** 2023-02-17

**Authors:** Xue Liu, Huiting Liu, Cailiang Gao, Wenbing Zeng

**Affiliations:** ^1^ PET-CT Center, Chongqing University Three Gorges Hospital, Chongqing, China; ^2^ Department of radiology, Chongqing University Three Gorges Hospital, Chongqing, China

**Keywords:** ^68^Ga-FAPI, ^18^F-FDG, fibroblast activating protein, abdominal and pelvic malignancy, meta-analysis, PET/CT

## Abstract

**Purpose:**

The purpose of this study is to compare the application value of ^68^Ga-FAPI and ^18^F-FDG PET/CT in primary and metastatic lesions of abdominal and pelvic malignancies (APMs).

**Materials:**

The search, limited to the earliest available date of indexing through 31 July 2022, was performed on PubMed, Embase, and Cochrane Library databases using a data-specific Boolean logic search strategy. We calculated the detection rate (DR) of ^68^Ga-FAPI and ^18^F-FDG PET/CT in the primary staging and recurrence of APMs, and pooled sensitivities/specificities based on lymph nodes or distant metastases.

**Results:**

We analyzed 473 patients and 2775 lesions in the 13 studies. The DRs of ^68^Ga-FAPI and ^18^F-FDG PET/CT in evaluating the primary staging and recurrence of APMs were 0.98 (95% CI: 0.95-1.00), 0.76 (95% CI: 0.63-0.87), and 0.91(95% CI: 0.61-1.00), 0.56 (95% CI: 0.44-0.68), respectively. The DRs of ^68^Ga-FAPI and ^18^F-FDG PET/CT in primary gastric cancer and liver cancer were 0.99 (95% CI: 0.96-1.00), 0.97 (95% CI: 0.89-1.00) and 0.82 (95% CI: 0.59-0.97), 0.80 (95% CI: 0.52-0.98), respectively. The pooled sensitivities of ^68^Ga-FAPI and ^18^F-FDG PET/CT in lymph nodes or distant metastases were 0.717(95% CI: 0.698-0.735) and 0.525(95% CI: 0.505-0.546), and the pooled specificities were 0.891 (95% CI: 0.858-0.918) and 0.821(95% CI: 0.786-0.853), respectively.

**Conclusions:**

This meta-analysis concluded that ^68^Ga-FAPI and ^18^F-FDG PET/CT had a high overall diagnostic performance in detecting the primary staging and lymph nodes or distant metastases of APMs, but the detection ability of ^68^Ga-FAPI was significantly higher than that of ^18^F-FDG. However, the ability of ^68^Ga-FAPI to diagnose lymph node metastasis is not very satisfactory, and is significantly lower than that of distant metastasis.

**Systematic review registration:**

https://www.crd.york.ac.uk/prospero/, identifier CRD42022332700.

## 1 Introduction

In recent years, the incidence and mortality of cancer have increased. An estimated 1,918,030 new cancer cases and 609,360 cancer deaths are expected in the United States, as published on January 12, 2022 (1). In the abdominal and pelvic malignancies (APMs), the proportions of liver cancer (LC), gastric cancer (GC), pancreatic cancer, colorectal cancer, and female reproductive system tumors are relatively high (1). Therefore, extensive clinical and basic research to strengthen the mission of health, extend life expectancy, and reduce the burden of disease and disability is crucial. Early diagnosis and accurate evaluation of treatment decisions and prognoses are of great significance (2). Given this fact, researchers have been stepping up their efforts to address these clinical issues (3).

The tumor microenvironment (TME) is a complex and dynamic framework that plays a key role in the survival, proliferation, spread and drug resistance of malignant cells through tumorigenic signaling pathways (4–6). Recently, the cancer-promoting role of the TME has become an the issues of interest to scientists (3). The tumor stroma is the main component of tumor lesions and has common components among various types of cancer (3). In addition to affecting tumor cells, the TME also affects a variety of nonmalignant cells (including immune cells, endothelial cells, epithelial cells, fibroblasts, and adipocytes), which are coordinated through complex, dynamic networks of different cytokines and chemokines (5, 7). A series of previous studies have led to a shift in the current research focus and direction of drug development from “tumor” to TME elements, which has aroused researchers’ interest in potential molecular imaging applications and therapies (8). Cancer-associated fibroblasts (CAFs) are an extremely heterogeneous and plastic cell population with different sources, functions and surface markers, which exist in various types of malignant solid tumors and are highly expressed, closely related to tumor progression, invasion and metastasis, and have become an attractive target for the TME (6, 9). However, when it is not expressed or is underexpressed in the stroma of normal tissues and benign tumors (10), it can usually be identified by fibroblast activating protein (FAP) as a marker (11). FAP, a type II membrane-bound glycoprotein with dipeptidyl peptidase and endopeptidase activities, is highly expressed in the membranes and stroma of CAFs, especially in approximately 90% of epithelial tumors (10), such as liver, colorectal, ovarian, and pancreatic cancers (2, 12, 13). In this case, using FAP as a CAF identifier and designing FAP-specific PET radiotracers and therapeutic radioligands are some of the results of efforts over the years (3). Therefore, FAP is an important and promising target for cancer therapy (14). In recent years, FAP inhibitors (FAPIs) have become a new targeted molecular probe in nuclear medicine and have attracted much attention in cancer diagnosis and treatment. Currently, dozens of radiopharmaceuticals targeting FAP have been developed, such as FAPI-01, FAPI-2, FAPI-04, FAPI-42, FAPI-46, and FAPI-74 (15–18). In a recent study, high-quality images were obtained using gallium-68-FAPI-04 positron emission tomography/computed tomography (^68^Ga-FAPI-04 PET/CT) showing good biodistribution properties and a high tumor background ratio in 28 tumors, including abdominal and pelvic tumors (19).

Recent years have seen an explosion in publications on ^68^Ga-FAPI, and FAPI imaging has opened a new chapter in molecular imaging for tumors and nontumor (6). Several studies have demonstrated ^68^Ga-DOTA-FAPI to be useful for diagnosing and differentiating primary tumors, detecting metastases, and performing image-guided intervention (20–24). However, its clinical effects and indications are not fully established (6). Fluorine-18-fluorodeoxyglucose (^18^F-FDG) PET/CT is an important imaging tool for preoperative systematic evaluation, tumor staging, and analysis of the efficacy of tumor treatment (25). However, ^18^F-FDG PET/CT imaging has certain limitations for some tumors, such as gastric mucinous adenocarcinoma, well-differentiated hepatocellular carcinoma, and renal cell carcinoma (25). Recently, ^68^Ga-FAPI and ^18^F-FDG imaging in various tumors has been studied to confirm the advantages and disadvantages of the two methods. FAPI is considered a promising molecular imaging agent because both of these studies confirm that ^68^Ga-FAPI can assess the primary tumor stage and detect lymph nodes in addition to distant metastases better than ^18^F-FDG (24, 26–30). Due to different sample sizes, uneven quality, and geographical influences, these results exhibit a high heterogeneity. The authors of a meta-analysis published in 2021 concluded that ^68^Ga-FAPI PET imaging was good at diagnosing primary and distant metastases in tumors and non-tumors (3). However, the study included only a few articles and did not include many valuable new papers published after March 2021. The results should also be interpreted cautiously because they are based on a heterogeneous set of studies.

Therefore, to further evaluate which of ^68^Ga-FAPI and ^18^F-FDG PET/CT were better in tumors, the aim of our study was to compare the application of ^68^Ga-FAPI and ^18^F-FDG PET/CT in primary and metastatic lesions of APMs.

## 2 Materials and methods

This meta-analysis was in accordance with the Preferred Reporting Items for Systematic Reviews and Meta-Analyses (PRISMA) statement. This project was registered in the PROSPERO database (registration number: CRD42022332700).

### 2.1 Data sources and search strategy

We performed electronic literature searches of the PubMed, Embase, and Cochrane Library databases for English-language articles from the earliest available date of indexing through July 31, 2022. The search was performed using a data-specific Boolean logic search strategy using the following keywords: FAP, FAPI, fibroblasts, cancer-associated fibroblasts, CAF, PET, PET/CT, PET-CT, FDG, fluorodeoxyglucose, and positron emission tomography. To obtain more comprehensive search information, we also manually searched the reference lists of identified publications. The search process was performed independently by the two reviewers (XL and HTL).

### 2.2 Inclusion and exclusion criteria

Published articles that met the following conditions were included in the analysis.

(1) ^68^Ga-FAPI and ^18^F-FDG PET/CT were evaluated simultaneously as diagnostic methods for APMs (primary tumor, lymph node involvement, and distant metastasis). Abdominal and pelvic lesions refer to tumors of the liver, pancreas, gallbladder, spleen, gastrointestinal tract, urinary system, female reproductive system and adrenal glands.
(2) The lesions were confirmed by histopathology or combined clinical/imaging follow-up.
(3) Sufficient data were provided to calculate the number of positive cases with respect to the primary APM tumor, or true-positive, false-positive, false-negative, and true-negative of non-primary tumors (lymph nodes or distant metastases).

The exclusion criteria were as follows: (1) overlapping papers; (2) review articles, animal experiments, editorials or letters, comments, and conference proceedings; (3) a lack of access to the full text; and (4) a sample size of fewer than 10 patients or lesions.

### 2.3 Quality assessment

Two reviewers (XL and HTL) independently evaluated each eligible article’s methodological quality. Any disagreements were resolved through consultation or intervention by the third reviewer. The evaluation is based on the modified Quality Assessment of Diagnostic Accuracy Studies version 2 (QUADAS-2), as recommended by the Cochrane Collaboration (31). Each item was evaluated as “high”, “low”, or “unclear”.

### 2.4 Data extraction

Data extraction was carried out for the remaining articles that met the criteria. For each study, we extracted the following data: first author name, year of publication, country, study design (prospective, retrospective), type of APM, diagnostic criteria, imaging purpose, image interpretation, age, sex, sample size, PET/CT scan range, type of imaging agent, injection activity, interval between the FAPI and FDG scans, maximum standardized uptake value (SUVmax) and tumor-to-background ratio (TBR) of the primary lesion, type of image analysis (qualitative, quantitative or semiquantitative), adverse events of imaging agents.

We recorded or calculated the specificity (SEN), sensitivity (SPE), and accuracy per patient and per lesion. When literature evaluation included multiple malignancies such as those of the neck, chest and abdomen, we only extracted data from abdominal tumors. If the abdominal tumor had fewer than 10 patients or lesions, the article was abandoned. When both primary and non-primary tumors (metastases) were evaluated, these data were collected for subgroup analysis. The authors were not contacted to retrieve unpublished data. Data were cross-checked and any discrepancies were discussed to reach a consensus (XL, HTL and CLG).

### 2.5 Statistical analysis

This study collected data for each eligible study. Descriptive statistics and frequency tables were used to summarize the data. The analysis was performed with subgroups of primary and non-primary tumors, and diagnostic pooled assessments of ^68^Ga-FAPI and ^18^F-FDG PET/CT were performed in the subgroups. On a patient-level basis, we evaluated the value of ^68^Ga-FAPI and ^18^F-FDG PET/CT in primary tumors, including primary staging and recurrence. Non-primary tumors, including lymph node, peritoneum, liver, bone and other metastases, were evaluated at the lesion-based level. The primary objective of this study was to evaluate the application value of ^68^Ga-FAPI and ^18^F-FDG PET/CT in the primary staging and recurrence of APMs using the detection rate (DR). In addition, we separately evaluated the detection value of ^68^Ga-FAPI and ^18^F-FDG PET/CT in the primary staging of GC and LC. DR was defined as the ratio between the number of patients or lesions with at least one suspected lesion detected by the imaging facility and the total number of abdomen-pelvic malignancy patients who underwent the scan. The secondary objective of this study was to evaluate the SEN, SPE, positive likelihood ratio (PLR), negative likelihood ratio (NLR), and diagnostic odds ratio (DOR) of ^68^Ga-FAPI and ^18^F-FDG PET/CT in metastatic lesions of APMs. A bivariate normal random-effects model for measures was used to analyze and pool the summary points for sensitivity and specificity and their 95% confidence intervals (CIs). The hierarchical summary receiver-operating characteristic (SROC) model was used performed to draw SROC curves and calculate the area under the curve (AUC). I^2^ and Cochran’s Q homogeneity tests were used to evaluate the consistency of the data (the higher the inconsistency, the greater the uncertainty of the meta-analysis results). According to Higgins JPT et al. (32) in 2003, heterogeneity was divided into low, medium and high levels, expressed with I^2^ as 25%, 50% and 75%, respectively. Multiple factors may lead to heterogeneity bias, and no single value is recommended for further analysis. We defined low/medium heterogeneity as acceptable (i.e., I2<50%). In the case of significant heterogeneity between studies, subgroup analysis or meta-regression was performed to analyze the data to determine the source of heterogeneity. As described by Deeks and colleagues (33), we examined the possibility of publication bias by using an effective sample size funnel plot and a regression test of asymmetry. Tests for significance were two-tailed, with a statistically significant P value threshold of 0.05. All statistical analyses were carried out using Stata version 16.0 software (StataCorp LP, College Station, TX, USA), Review Manager software (Cochrane Collaboration, version 5.3.5, London, United Kingdom) and MetaDiSc 1.4 (Clinical Biostatistics team of the Ramón y Cajal Hospital in Madrid, Spain).

## 3 Results

### 3.1 Literature search and study selection

**Figure 1:**
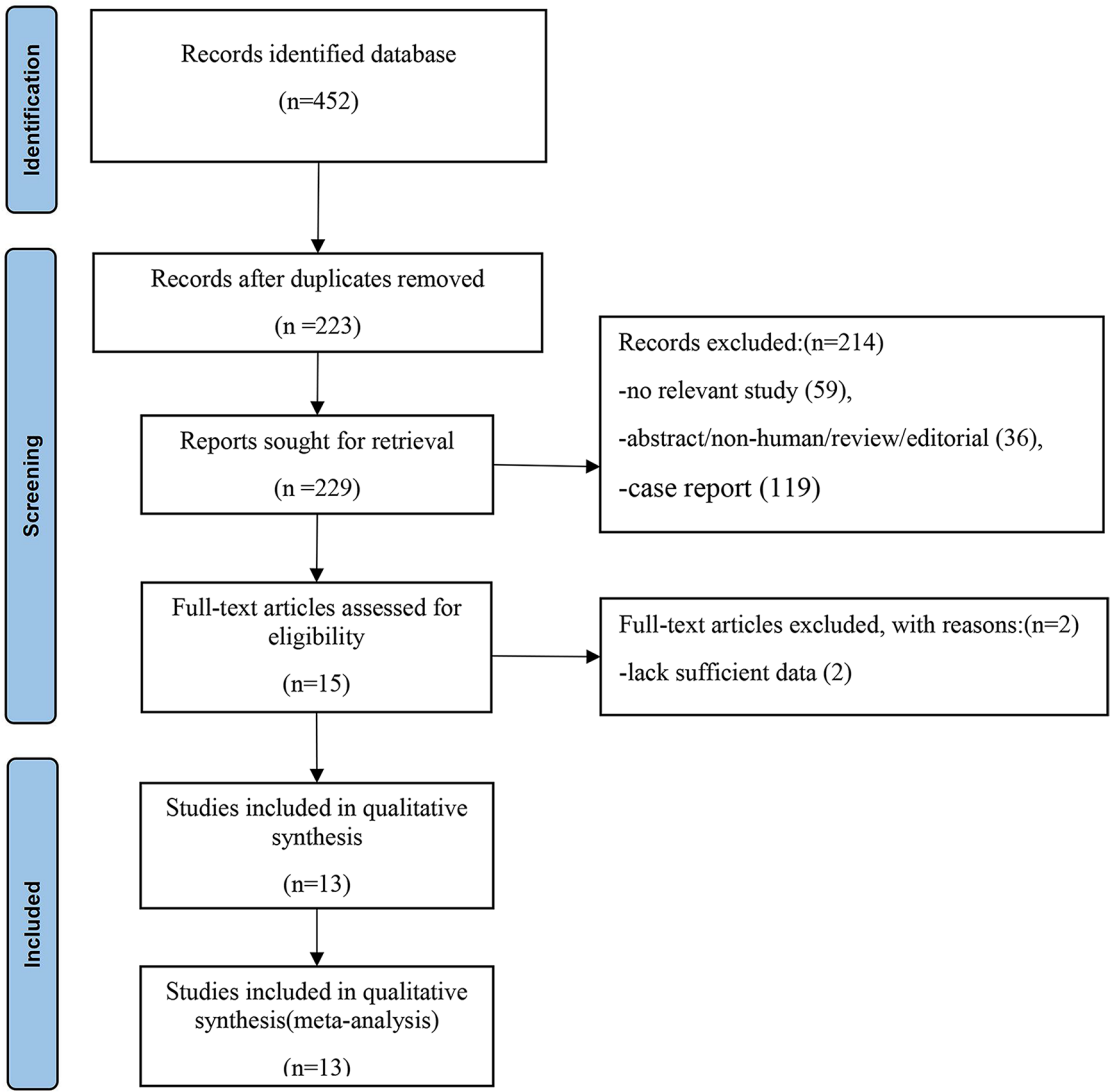
Flowchart of the search for eligible studies on ^68^Ga-PAFI and ^18^F-FDG PET/CT in patients of abdominal and pelvic malignancies. Thirteen articles were finally selected for this meta-analysis.

A total of 452 articles were retrieved from the PubMed/MEDLINE, Embase and Cochrane Library databases. Two hundred and twenty-three duplicate articles were excluded. Titles and abstracts were screened according to the established inclusion and exclusion criteria, 208 articles were deleted, leaving 15 papers, and a full-text search was conducted. Full-text reading was conducted, and 13 articles were finally eligible for meta-analysis. The detailed process of literature screening is shown in **Figure 1**.

### 3.2 Characteristics of the included studies

Overall, we analyzed 473 patients and 2775 lesions in the 13 studies (2, 12, 13, 17, 18, 24, 26–30, 34, 35). These studies were published between 2020 and 2022, among which 8 studies (2, 12, 17, 18, 24, 28, 30, 34) were from China, 3 studies (26, 27, 29) were from Turkey, and the others were from Thailand (13)and Israel (35). Eight studies (2, 12, 17, 24, 26, 27, 34, 35) had a prospective study design, and the remainder had a retrospective study design. Eight studies (13, 17, 18, 26–29, 34) assessed both primary staging and tumor recurrence, and five studies (2, 12, 24, 30, 35) assessed only primary staging. Although Chen et al. (17) and Komek et al. (26) evaluated the staging and recurrence of primary tumors in the original text, due to the limited sample size for the evaluation of primary tumors, we only extracted data on recurrence for analysis. One study (17) evaluated tumors in multiple parts of the body, including lung, esophageal, nasopharyngeal, colorectal, hepatic, gastric and ovarian cancer. However, we only extracted data from APM for analysis. All of the subjects included in this meta-analysis were APM, including 8 studies for gastrointestinal tumors (2, 17, 26–29, 34, 35), 4 for liver tumors (2, 13, 17, 24), 2 for pancreatic tumors (12, 29), and 1 for ovarian tumors (17). We found no other literature that simultaneously compared ^68^Ga-FAPI and ^18^F-FDG the female reproductive system (ovary, uterus, vagina), urinary system (kidney, prostate, bladder, ureter), adrenal gland, gallbladder, and spleen malignancies.

PET/CT was used as the imaging mode in all included studies. Seven studies (2, 12, 17, 26, 27, 29, 34) reported the PET/CT scanning scope, mostly from the head to the mid-upper thighs. The ^68^Ga-FAPI and ^18^F-FDG imaging scans were performed within a week of each other. ^68^Ga-FAPI-04 was employed in all studies except for that conducted by Siripongsatian et al. (13), who used the imaging agent ^68^Ga-FAPI-46. Fu et al. (18) used both imaging agents ^68^Ga-FDAPI-04 and ^18^F-DAPI-42 in their study.

All studies compared the SUVmax or TBR values of ^68^Ga-FAPI and ^18^F-FDG PET/CT in primary tumors, we found that FAPI-SUVmax was higher than FDG-SUVmax in most of them, and only the Komek et al. (26) study had a lower FAPI-SUVmax than FDG-SUVmax (mean: 11.54 vs. 18.93). All participants tolerated the ^68^Ga-FAPI PET/CT scan. No ^68^Ga-FAPI-related pharmacological effects or physiological responses occurred (12, 17, 28, 35). Furthermore, the authors of all the articles declared no conflicts of interest. The main characteristics of the 13 studies included in the meta-analysis are shown in **Tables 1**, **2**.

**Table 1:** Basic study and patient characteristics.

Author	Year	Country	Gender (male/female)	Age (years)	Imaging purpose	Patients/Lesions (N)	Study design	Tumor type	Imaging analyses	Blind	Diagnostic criteria
Chen et al. (17)	2020	China	47/28	Median=61.5	Initial staging, relapsed	Relapsed (12)	P	Colorectal ADC,Liver cancer, Gastric cancer, Ovarian cancer	V+Q	Yes	HP
Fu et al. (18)	2022	China	37/24	Median=57	Initial staging, relapsed	Initial staging (61) Metastasis (146)	R	Gastric cancer	V+S	Yes	Laparoscopic exploration or HP or Ascites cytology
Guo et al. (2)	2021	China	25/9	Mean=60.6	Initial staging	Initial staging (23) Metastasis (190)	P	Liver cancer	V+Q	Yes	HP
Gündoğan et al. (27)	2022	Turkey	12/9	Median=61	Initial staging, relapsed	Initial staging (15) Metastasis (522)	P	Gastric ADC	V+Q	NG	HP
Kuten et al. (35)	2022	Israel	6/7	Median=70	Initial staging	Initial staging (10)	P	Gastric ADC	V+Q	NG	HP
Lin et al. (34)	2022	China	40/16	Mean=63.8	Initial staging, relapsed	Initial staging (45), Relapsed (11), Metastasis (862)	P	Gastric cancer	V+S	NG	HP
Pang et al. (28)	2021	China	18/17	Median=64	Initial staging, relapsed	Initial staging (19), Relapsed (16), Metastasis (306)	R	Gastric, duodenal, and colorectal cancers	V+Q	Yes	HP
Pang et al. (12)	2022	China	25/11	Median=60	Initial staging	Initial staging (36), Metastasis (333)	P	Pancreatic cancer	V+S	Yes	HP, imaging FU
Shi et al. (24)	2021	China	18/2	Mean=58.0	Initial staging	Initial staging (20), Metastasis (23)	P	Liver cancer	V+Q	NG	HP, imaging FU
Siripongsatian et al. (13)	2022	Thailand	21/6	Median=68	Initial staging, relapsed	Initial staging (21), Relapsed (15), Metastasis (76)	R	Liver cancer	V+Q	Yes	FP, MRI
Wang et al. (30)	2021	China	24/1	Mean=59.40	Initial staging	Initial staging (25), Metastasis (35)	R	Hepatocellular carcinoma	V+Q	Yes	HP
Şahin et al. (29)	2021	Turkey	19/12	Mean=61.9	Initial staging, relapsed	Initial staging (31), Metastasis (98)	R	Colorectal cancer, Pancreas cancer, Gastric cancer, Other	V+Q	NG	HP, imaging FU, tumor biomarker
Kömek et al. (26)	2022	Turkey	22/17	Mean=61	Initial staging, relapsed	Relapsed (36), Metastasis (184)	P	Colorectal cancer	V+Q	NG	HP

P, prospective; R, retrospective; Ca, cancer; ADC, ADC; CCC, cholangiocarcinoma; HCC, hepatocellular carcinoma; NG, not given; V, visual analysis; Q, quantitative analysis; S, semi-quantitative analysis; HP, Histopathology; FU, follow-up; FDG, fluorodeoxyglucose.

**Table 2:** Technical aspects of ^68^Ga-FAPI and ^18^F-FDG in the included studies.

Authors	PET/CT scanner	Radiotracer 1 (Activity)	Radiotracer 2 (Activity)	Time interval between the two scans	Scanning scope	PC SUV-max Mean/Median		PC TBR Mean/Median	
						FAPI	FDG	FAPI	FDG
Chen et al. (17)	Discovery MI, GE Healthcare	^68^Ga-FAPI-04 (1.8–2.2 MBq)	^18^F-FDG (3.7 MBq/kg)	Within 7 days	From the head to the upper thighs	16.18 (7.24–25.47)	3.34 (2.08–10.7)	NG	NG
Fu et al. (18)	Biograph mCTx scanner (Siemens Healthcare) and uEXPLORER (United Imaging)	^68^Ga-FAPI-04/^18^F-FAPI-42 (NG)	^18^F-FDG (NG)	Within 1 week	NG	14.60 (3.00–30.90)	4.35 (1.70–21.70)	11.04 (2.69–27.13)	2.81 (1.06–16.00)
Guo et al. (2)	Discovery MI, GE Healthcare	^68^Ga-FAPI-04 (148–259 MBq)	^18^F-FDG (3.7 MBq/kg)	Within 7 days	From the head to the upper thighs	13.61 (4.66–23.21)	4.24 (2.63–11.26)	5.55 (1.05–10.62)	1.17 (0.89–4.41)
Gündoğan et al. (27)	Discovery IQ 4 ring 20 cm axial FOV, GE Healthcare	^68^Ga-FAPI-04 (2 MBq/kg)	^18^F-FDG (3.5–5.5 MBq/kg)	Maximum 1 week apart	From the vertex to mid-thigh	11.0 (0.8-25.1)	6.1 (2.2-24.6)	8.8 (2.4-27.0)	5.1 (2.4-33.7)
Kuten et al. (35)	Discovery MI, GE Healthcare	^68^Ga-FAPI-04 (1.8–2.2MBq/kg)	^18^F-FDG (3.7 MBq/kg)	6 days (range 1–23 days)	NG	15.9 (4–32)	5.5 (1.6–32)	11.9 (2.2–23.9)	3.2 (0.8–9.7)
Lin et al. (34)	Care Dose 4D (Biograph mCT64, Siemens Healthcare)	^68^Ga-FAPI-04 (111–185MBq)	^18^F-FDG (3.7 MBq/kg)	Less than 1 week	From the head to the upper thighs	10.3 ± 3.8	8.1 ± 4.9	11.6 ± 5.4	5.8 ± 3.6
Pang et al. (28)	Discovery MI, GE Healthcare	^68^Ga-FAPI-04 (1.8–2.2 MBq/kg)	^18^F-FDG (3.7 MBq/kg)	2 days (1–6 days)	NG	15.9 (12.2–21.3)	7.9 (7.1–14.9)	NG	NG
Pang et al. (12)	Discovery MI, GE Healthcare	^68^Ga-FAPI-04 (1.8–2.2 MBq/kg)	^18^F-FDG (3.7 MBq/kg)	2 days (range, 1–6 days)	From the head to the upper thighs	8.6 (2.9–18.4)	2.7 (1.0–6.8)	NG	NG
Shi et al. (24)	PoleStar m660, Sinounion Healthcare	^68^Ga-FAPI-04 (3.59 ± 0.47 MBq/kg)	^18^F-FDG (3.7 MBq/kg)	Within 3 days	NG	8.47 ± 4.06	4.86 ± 3.58	7.13 ± 5.52	2.39 ± 2.21
Siripongsatian et al. (13)	64-slice Siemens Biograph vision scanner	^68^Ga-FAPI-46 (2.59MBq/kg)	^18^F-FDG (2.59 MBq/kg)	Within 1 week	NG	24.02 (19.82–26.00)	8.66 (4.17–23.23)	21.07 (17.39–23.94)	3.12 (1.62–7.74)
Wang et al. (30)	FAPI: mMI510, Union imaging FDG : Biograph mCT Flow scanner, Siemens	^68^Ga-FAPI-04 (185 MBq)	^18^F-FDG (NG)	1day	NG	6.96 ± 5.01	5.89 ± 3.38	11.90 ± 8.35	3.14 ± 1.59
Şahin et al. (29)	GE Healthcare	^68^Ga-FAPI-04 (2–3 MBq/kg)	^18^F-FDG (5 MBq/kg)	NG	From the vertex to the upper part of the femur	7.8 (2.3–13.7)	5.0 (4.3–10.2)	5.2 (2.8–10.4)	1.5 (1.2–3.4)
Kömek et al. (26)	GE Healthcare	^68^Ga-FAPI-04 (2MBq/kg)	^18^F-FDG (3.5–5.5 MBq/kg)	1–6 days	From the vertex to mid-thigh	11.54 ± 4.74	18.93 ± 10.14	15.14 ± 10.31	10.22 ± 5.8

FAPI, fibroblast activation protein inhibitor; FDG, fluorodeoxyglucose; NG, Not given; PC, primary tumour; SUV-max, maximum standardized uptake value; TBR, tumor-to-background ratio; GE, General Electric Company.

### 3.3 Risk of bias and applicability

**Figure 2:**
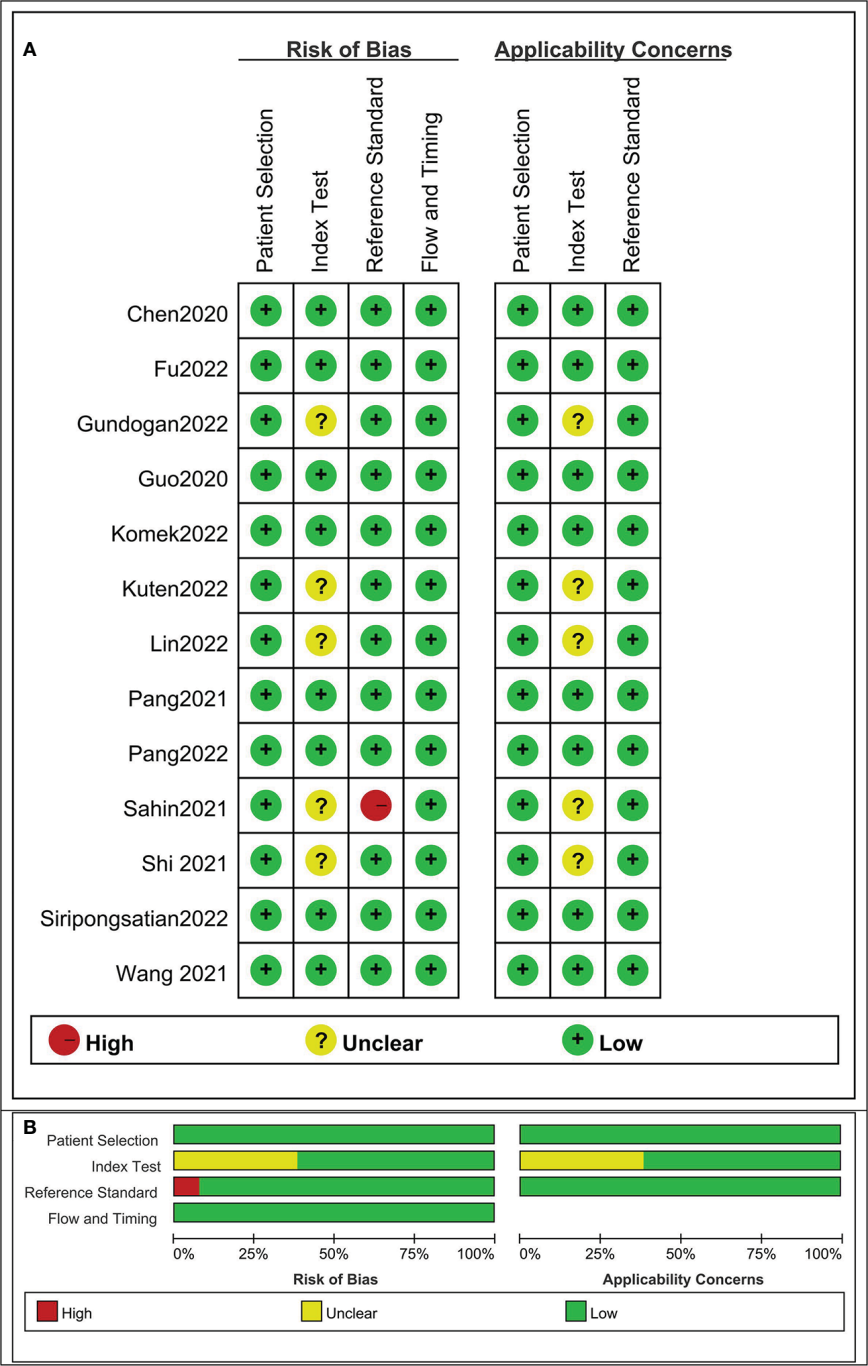
Risk of bias and applicability concerns the summary **(A)** and graph **(B)** of the studies included in the systematic review according to the QUADAS-2 tool. Overall quality of the included studies was deemed satisfactory.

The risk of bias and applicability concerns for the included studies were assessed using QUADAS-2 (**Figure 2**). None of the studies were of low quality, and the overall quality of the studies was satisfactory.

### 3.4 Quantitative analysis (meta-analysis)

#### 3.4.1 Based on primary tumor performance analysis

**Figure 3:**
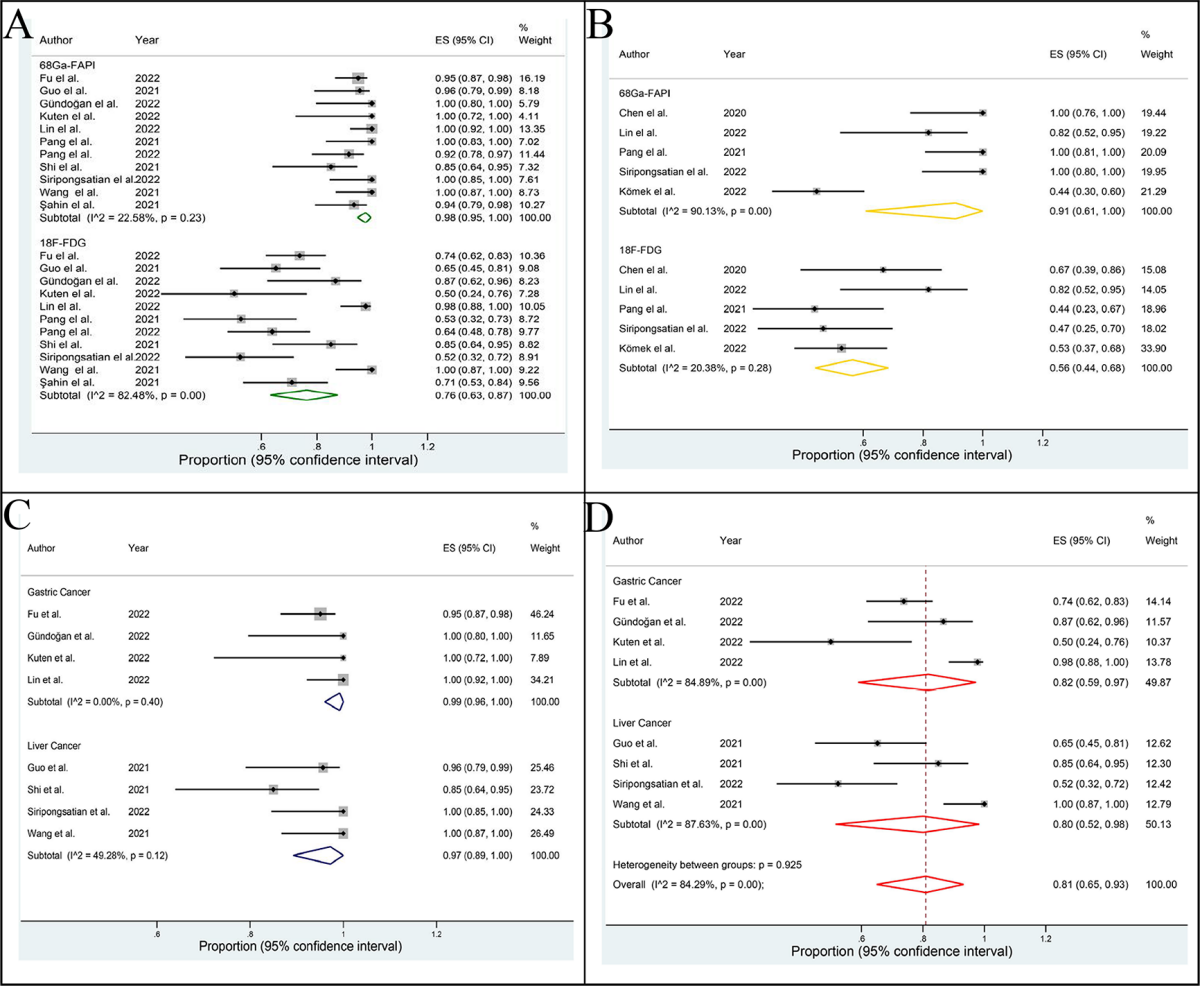
Forest plot of this meta-analysis. The detection rates of ^68^Ga-PAFI and ^18^F-FDG PET/CT in evaluating the primary staging **(A)** and recurrence **(B)** of abdominal and pelvic malignancy. The detection rates of ^68^Ga-FAPI **(C)** and ^18^F-FDG **(D)** PET/CT in evaluating the primary gastric cancer and liver cancer.

The DRs of ^68^Ga-FAPI and ^18^F-FDG PET/CT in evaluating the primary staging of APM were 0.98 (95% CI: 0.95-1.00; I^2^= 22.58%, p=0.23) and 0.76 (95% CI: 0.63-0.87; I^2^= 82.48%, p=0.00), respectively (**Figure 3A**). The difference between the two groups was statistically significant (P =0.00).

The DRs of ^68^Ga-FAPI and ^18^F-FDG PET/CT in identifying recurrence of APM were 0.91 (95% CI: 0.61-1.00; I^2^= 90.13%, p=0.00) and 0.56 (95% CI: 0.44 0.68; I^2^= 20.38%, p=0.28), respectively (**Figure 3B**). The difference between the two groups was statistically significant (p=0.04).

The DRs of ^68^Ga-FAPI and ^18^F-FDG PET/CT in primary GC and LC were 0.99 (95% CI: 0.96-1.00; I^2^= 0.00%, p=0.40), 0.97 (95% CI: 0.89-1.00; I^2^= 49.28%, p=0.12) and 0.82 (95% CI: 0.59-0.97; I^2^= 84.89%, p=0.00), 0.80 (95% CI: 0.52-0.98; I^2^= 87.63%, p=0.00), respectively (**Figures 3C, D**). Due to the limited sample size, we did not assess the DR of ^68^Ga-FAPI and ^18^F-FDG PET/CT in recurrence of GC and LC.

#### 3.4.2 Based on non-primary tumor performance analysis

**Figure 4:**
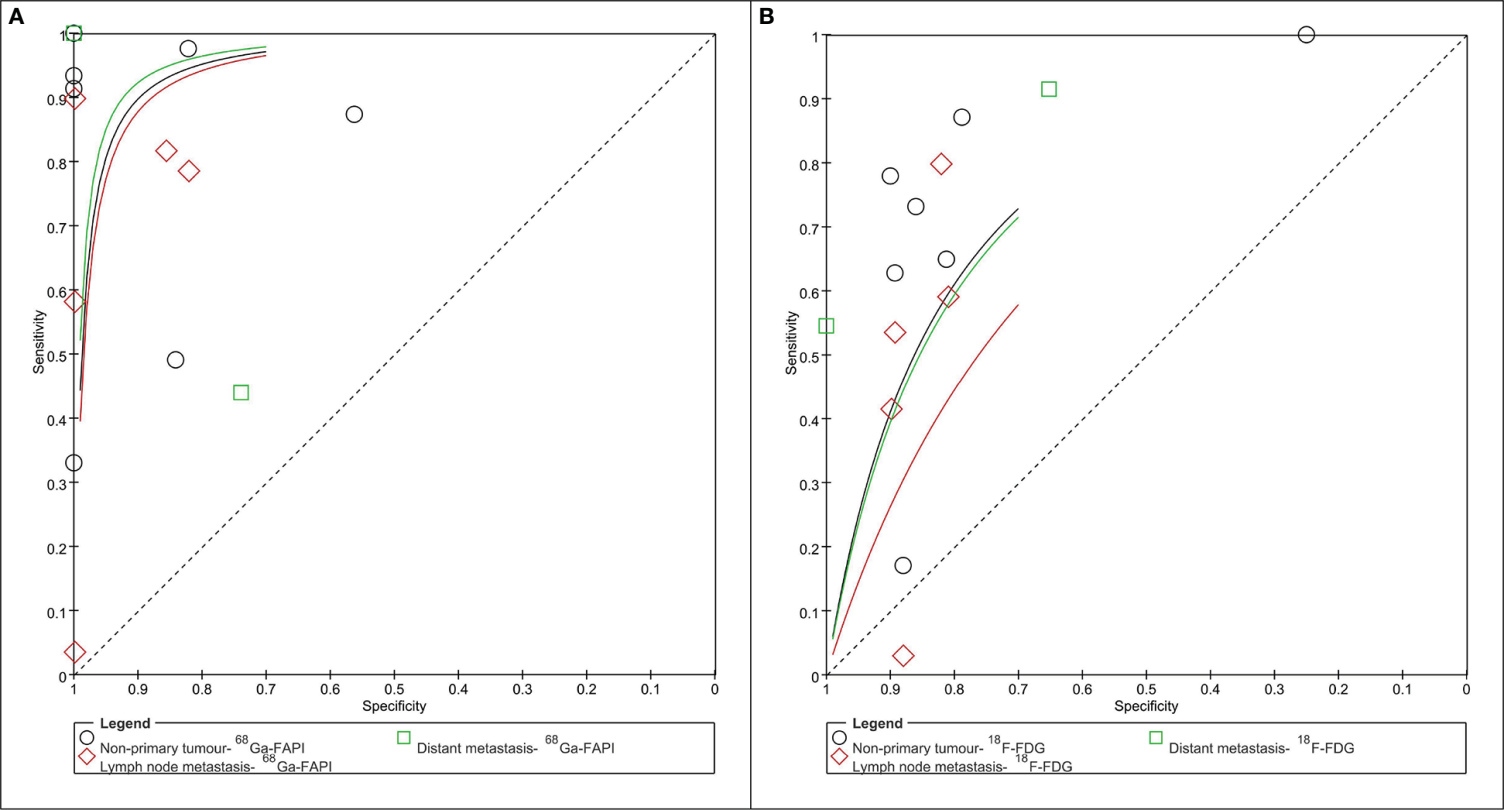
Summary receiver operator characteristic graph of ^68^Ga-PAFI **(A)** and ^18^F-FDG **(B)** PET/CT for non-primary tumor (lymph node and/or distant metastasis).

The pooled SENs of ^68^Ga-FAPI and ^18^F-FDG PET/CT in non-primary tumors were 0.717 (95% CI: 0.698-0.735; I^2^= 99.1%, p=0.000) and 0.525 (95% CI: 0.505-0.546; I^2^= 98.5%,p=0.000), and the pooled SPEs were 0.891 (95% CI: 0.858-0.918; I^2^= 83.0%, p=0.000) and 0.821 (95% CI: 0.786-0.853; I^2^= 64.4%, p=0.00), respectively. The AUCs were 0.946 and 0.841, respectively (**Figure 4**).

##### 3.4.2.1 Based on lymph node metastasis performance analysis

The pooled SEN, SPE, and DOR of ^68^Ga-FAPI PET/CT in the assessment of lymph node metastases were 0.421 (95% CI: 0.389-0.453; I^2^= 99.4%, p=0.000), 0.908 (95% CI: 0.874-0.935; I^2^= 82.6%, p=0.000) and 35.860 (95% CI: 11.320-113.61; I^2^= 44.7%, p=0.093), respectively. The pooled SEN, SPE, and DOR of ^18^F-FDG PET/CT in the assessment of lymph node metastases were 0.235 (95% CI: 0.207-0.264; I^2^= 98.3%, p=0.000), 0.837 (95% CI: 0.799-0.870, I^2^= 0.0%, p=0.573) and 3.257 (95% CI: 0.656-16.176; I^2^= 90.1%, p=0.000), respectively. The AUC of ^68^Ga-FAPI and ^18^F-FDG PET/CT were 0.938 and 0.877, respectively (**Figure 4**).

##### 3.4.2.2 Based on distant metastasis performance analysis

The pooled SEN, SPE, and DOR of ^68^Ga-FAPI PET/CT in the assessment of distant metastasis were 0.918 (95% CI:0.900-0.933; I^2^= 98.2%, p=0.000), 0.844 (95% CI: 0.729-0.924; I^2^= 52.6%, p=0.049) and 72.059 (95% CI:5.636-921.25; I^2^= 73.1%, p=0.001), respectively. The pooled SEN, SPE, and DOR of ^18^F-FDG PET/CT in the assessment of distant metastasis were 0.714 (95% CI:0.686-0.741; I^2^= 95.1%, p=0.000), 0.811 (95% CI: 0.691-0.900; I^2^= 62.0%, p=0.015), and 13.431 (95% CI: 5.759-31.322; I^2^= 0.0%, p=0.495), respectively. The AUC of ^68^Ga-FAPI and ^18^F-FDG PET/CT were 0.850 and 0.777, respectively (**Figure 4**).

### 3.5 Publication bias

**Figure 5:**
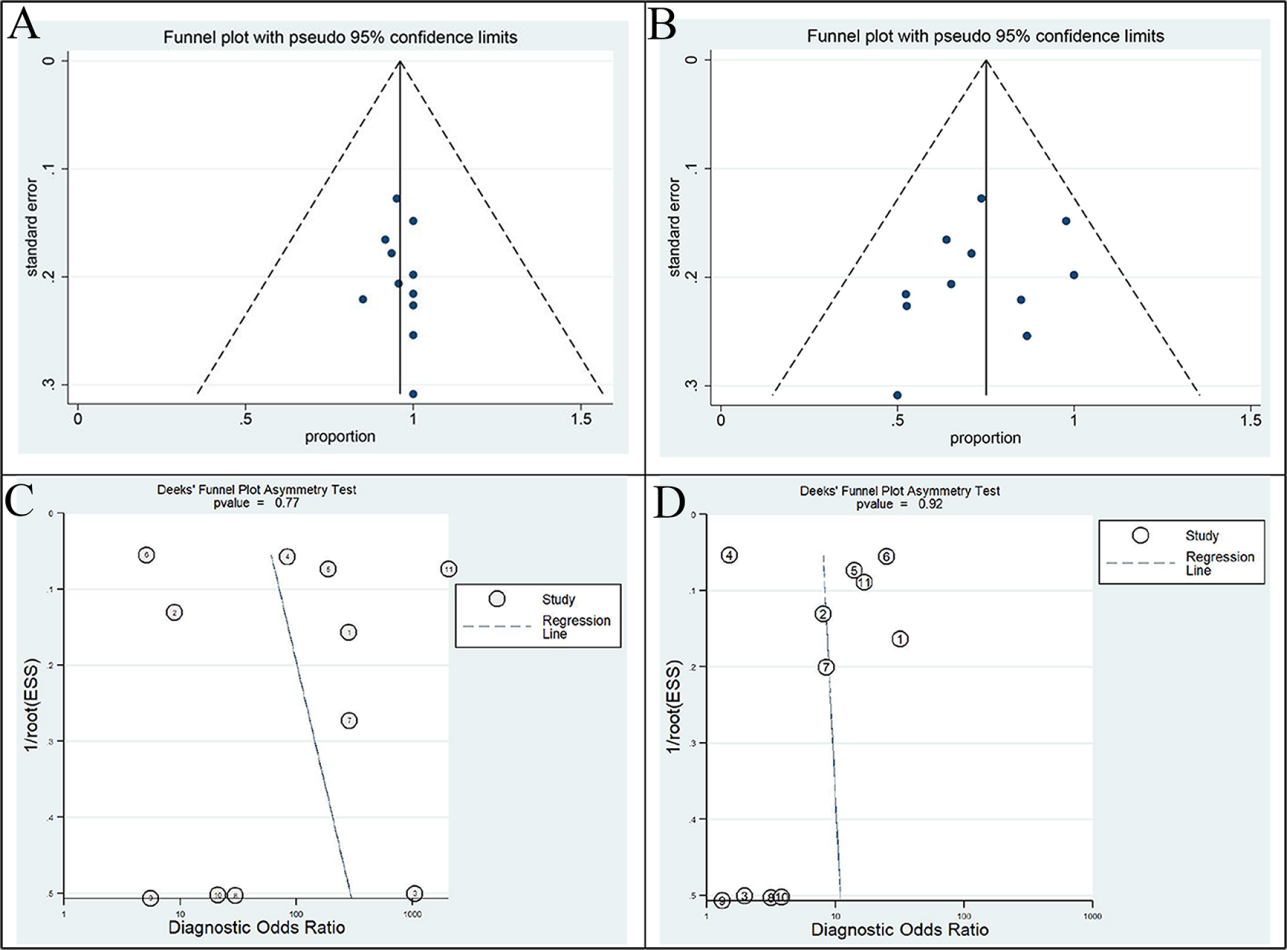
Funnel plot of this meta-analysis. Funnel plots with Egger’s test for ^68^Ga-FAPI **(A)** and ^18^F-FDG PET/CT **(B)** in primary tumor staging (all p>0.05). Funnel plots with Deek’s test for ^68^Ga-FAPI **(C)** and ^18^F-FDG PET/CT **(D)** in non-primary tumour (all p>0.05). All of these results indicate the absence of a publication bias.

Egger’s regression intercepts for DR pooling of ^68^Ga-FAPI and ^18^F-FDG PET/CT in primary tumor performance analysis were 0.317 (95% CI: -0.57 to 0.86, p=0.664) and 1.14 (95% CI: -3.68 to 1.47, p=0.358), respectively, indicating that publication bias was absent. Moreover, the funnel plots for both modalities were symmetric (**Figures 5A, B**). Analysis of non-primary tumors by ^68^Ga-FAPI and ^18^F-FDG PET/CT according to the linear regression detection method suggested regression coefficients of 3.52 (p=0.77) and 0.69 (p=0.92), respectively, indicating that there was no publication bias in the included studies (**Figures 5C, D**).

## 4 Discussions

This is the first study to conduct a head-to-head comparison of ^68^Ga-FAPI with ^18^F-FDG PET/CT in APM using a meta-analysis. Based on our results, ^68^Ga-FAPl performed better than ^18^F-FDG in APM primary staging, with DRs of 98% and 70%, respectively. There was no interstudy heterogeneity, indicating that our results were stable and reliable. A previous meta-analysis included the assessment of various cancers, including glioblastoma, head and neck tumors, and nasopharyngeal carcinoma, and the researches believed that the sensitivity of ^68^Ga-FAPI in identifying primary tumors was 100% (95%CI: 98%-100%) (6). In addition, when they analyzed abdominal tumors as a subgroup, the sensitivity ^68^Ga-FAPI was 100% for primary tumors and 87% for non-primary tumors, indicating the high diagnostic efficacy of this molecule.

In addition, this meta-analysis also evaluated the application value of ^68^Ga-FAPI and ^18^F-FDG in disease recurrence in APM, and the combined DR was 91% and 56%, respectively. In a retrospective study, the authors analyzed 16 patients with recurrent gastrointestinal tumors (28). They found that the positive rates of ^68^Ga-FAPI-04, ^18^F-FDG PET/CT and conventional evaluation were 100%, 57.1%, and 33.3%, respectively. Siripongsatian et al. (13) reported that the uptake-positive rates in locally recurring and residual tumor lesions were 46.7% (7/15) on ^18^F-FDG PET/CT and 100% (15/15) on ^68^Ga-FAPI PET/CT. Compared with the ^18^F-FDG-based TNM staging system, the ^68^Ga-FAPI-based TNM staging system was upgraded in 6 patients (6/23, 26.1%), resulting in management changes in 2 patients (2/23, 8.7%) (12). Their results also indicated that ^68^Ga-FAPI PET/CT was superior to ^18^F-FDG in detecting primary and metastatic lesions. Researchers in Thailand (13) and China (28) showed that ^68^Ga-FAPI PET/CT was more sensitive than ^18^F-FDG in the identification of liver and gastrointestinal primary tumors (100% vs. 52% and 100% vs. 53%). It seems that all published studies thus far support the evidence that ^68^Ga-FAPI PET/CT has a higher detection value than ^18^F-FDG for primary tumors, and our meta-analysis results indicate the same. This finding is mainly attributed to the excellent biodistribution characteristics of FAPIs, which can provide a better TBR and yield detailed anatomical maps (34). In addition to the higher tracer uptake of ^68^Ga-FAPI, the superior performance of ^68^Ga-FAPI PET/CT includes its enhanced ability to detect small metastases (diameter<1.0 cm). Tumor lesions >1-2 mm require the formation of a supportive stroma, and since the stroma volume may be larger than the tumor volume, stromal targeted PET imaging may be more sensitive than glycolytic targeted PET imaging in detecting small lesions (2).

All studies compared SUVmax or TBR values in the primary tumor. We found that the vast majority of ^68^Ga-FAPI values were higher than those of ^18^F-FDG, and only the Komek et al. (26) study had a lower FAPI-SUVmax than FDG-SUVmax (mean: 11.54 vs. 18.93) but failed to demonstrate a significant difference in terms of TBR. The possible reason is that the researchers evaluated patients with colorectal cancer, and hemorrhoid lesions reaching the anal canal showed a higher ^18^F-FDG than ^68^Ga-FAPI uptake. Hemorrhoids may show increased radioactivity concentration on ^18^F-FDG PET/CT but their SUVmax is lower on ^68^Ga-FAPI PET/CT than on ^18^F-FDG PET/CT (26). The cause of abnormal FAPI concentrations in hemorrhoids may be associated with mild fibrous tissue hyperplasia due to inflammation of various veins and the anal canal (36). Whether the high sensitivity and specificity of FAPI for tumor stroma confer any clinical value beyond a numerical advantage in the TBR is still unknown (26).

Our results showed that the DRs of the imaging agent ^68^Ga-FAPI in GC and LC were 99% and 97%, respectively, which were higher than those of the imaging agent ^18^F-FDG (82% and 80%). In Israel’s (35) small cohort study, ^68^Ga-FAPI was superior to ^18^F-FDG in detecting primary GC, with a DR of 100%, while that of ^18^F-FDG was only 50%. This shows that the high DR of ^68^Ga-FAPI is mainly due to the degree GC of differentiation and the known limitations of ^18^F-FDG in examining several GC subtypes, such as mucinous adenocarcinoma, noninterstitial diffuse carcinoma, and signed-ring cell carcinoma, raising the possibility that ^68^Ga-FAPI can be used as a radiotracer of choice in the evaluation of GC. In addition, the physiological uptake of ^18^F-FDG by the gastric wall also further limits the application of ^18^F-FDG PET/CT in the detection of GC. The results of Pang et al. (28) showed that ^68^Ga-FAPI PET/CT can be used to analyze different types of GC and thus may play a complementary role in resolving the uncertain results of ^18^F-FDG PET/CT. Lin et al. (34) also suggested that the lesions of signet-ring cell carcinoma were positive for ^68^Ga-FAPI and negative for ^18^F-FDG. Studies have reported a low FDG uptake in signet-ring cell carcinoma and mucinous carcinoma than in conventional adenocarcinoma, which may be due to the low expression of glucose transporter 1 (37–39).

Similarly, ^68^Ga-FAPI PET/CT is superior to ^18^F-FDG PET/CT in identifying liver lesions, which may improve the staging and subsequent treatment of LC. Guo et al. (2) suggested that ^68^Ga-FAPI-04 PET/CT can detect 96% (22/23) of primary liver tumors, with good contrast between the tumor and background, comparable to the DR of contrast-enhanced CT (96%) and liver MRI (100%). In contrast, ^18^F-FDG detected only 65% (15/23) of primary liver tumors. The study of Siripongsatian et al. (13) reported that 100% (21/21) of intrahepatic tumors were detected by ^68^Ga-FAPI, whereas only 52% (11/21) were detected by ^18^F-FDG. These results may be due to the higher uptake of FAPI by primary tumors and the lower hepatic background activity of FAPI compared with ^18^F-FDG. For hepatocellular carcinoma (HCC) with low expression of glucose transporter-1 and high expression of glucose-6-phosphatase, 40% of such HCC lesions appeared nonavid on FDG PET images (40). The tumor-to-nontumour liver uptake ratio of the well-differentiated HCC was approximately 1.1, indicating that ^18^F-FDG PET imaging is difficult to distinguish between the uptake of well-differentiated HCC lesions and healthy liver tissues (41). In addition, investigators observed that ^68^Ga-FAPI-04 uptake was higher in most primary intrahepatic cholangiocarcinoma lesions than in HCC lesions (2). This finding may be attributed to the fact that intrahepatic cholangiocarcinoma is a particular type of fibroproliferative tumor and because the number of CAFs tends to significantly exceed that of actual cholangiocarcinoma cells (42). The severity of the corresponding pathological grade of the primary tumor was positively correlated with the ^68^Ga-FAPI-04 uptake activity of the lesion (2). Therefore, ^68^Ga-FAPI can be useful in assessing the extent of disease and differentiating benign from malignant lesions, especially when assessment is difficult with ^18^F-FDG or conventional imaging. In view of the above discussion and analysis, ^68^Ga-FAPI seems to be a promising imaging model that may replace ^18^F-FDG for evaluation of abdominal malignancies.

Although high uptake of ^68^Ga-FAPI helps to improve lesion identification, it may lead to a higher false-positive rate. Guo et al. (2) reported 3 false-positive cases caused by ^68^Ga-FAPI, including 1 pulmonary inflammatory granuloma, 1 pulmonary infection, and 1 thyroid adenoma, which also showed high uptake on ^18^F-FDG. In addition, there were 4 cases of high ^68^Ga-FAPI uptake due to postoperative infection, which was mistaken as an indication of tumor recurrence. Nonspecific fibrosis induced by inflammation may contribute to the positive uptake of ^68^Ga-FAPI-04 (28, 43, 44). False-positive uptake of ^68^Ga-FAPI has been observed in inflammatory diseases (e.g., uteritis and abscesses), granulomatous diseases (e.g., tuberculosis), and other diseases in which the fibrotic response is activated (e.g., myelofibrosis and cirrhosis) (28). Thus, ^68^Ga-FAPI PET/CT might be problematic when differentiating between residual and/or recurrent disease and postradiation and/or postoperative inflammatory reactions (43).

In addition to comparing ^68^Ga-FAPI and ^18^F-FDG in the primary staging and recurrence of APM, this study also evaluated their efficacy in non-primary tumors. In non-primary tumors, ^68^Ga-FAPI had a higher SEN, SPE, DOR, and AUC than ^18^F-FDG. However, the various effect indicators showed a high level of heterogeneity (all P <0.05). Consequently, lymph nodes and distant metastases were subgroup analyzed to improve performance and heterogeneity. From our combined results, ^68^Ga-FAPI outperforms ^18^F-FDG in all aspects. It is important to note, however, that the pooled SEN of these two types of imaging agents in evaluating lymph nodes is generally unsatisfactory, with effect sizes less than 50%. In the study by Fu et al. (18), the coincidence rates of ^68^Ga-FAPI and ^18^F-FDG in lymph node staging were 50% and 45.4%, respectively, compared with pathology. ^68^Ga-FAPI and ^18^F-FDG PET/CT had the same low SEN (58.3% vs. 41.7%) and moderate DOR (77.3% vs. 63.6%), although the SPE was high (100% vs. 90%) in their study. In comparison with ^18^F-FDG, ^68^Ga-FAPI-04 PET detected more suspicious lymph node lesions but did not improve lymph node staging clinically (18). Gundoğan et al. (27) found that the SEN and SPE of ^68^Ga-FAPI-04 PET/CT in detecting lymph node metastasis were 100% and 95.2%, respectively, while those of ^18^F-FDG PET/CT were 71.4% and 93.7%, respectively. In the study of Lin et al. (34), ^68^Ga-FAPI PET/CT found only 20 true positives in 625 resected lymph nodes, with a calculated SEN of 19.2%. Therefore, FAPI has a strong ability to exclude lymph node metastases but has an unstable and limited ability to detect lymph node metastases. Its expression has been associated with multiple factors, such as local tumor invasion, lymph node metastasis, and poor prognosis, including tumor invasion, metastasis, and angiogenesis (8). Therefore, the high variability of ^68^Ga-FAPI in lymph node stage assessment (pooled SEN 38.9%-45.3%) may be unexpected, especially given that the lymph nodes usually consist of mesh cell networks of the fiber layer (6). Its relatively low performance in detecting lymph node metastasis may be related to the biological characteristics of the cancer and the degree of lymph node cell enrichment (6). It has been suggested that reflective isotopes used for FAPI labeling may affect the image resolution and thus the detectability of smaller tumor aggregates in lymph nodes, because the ^68^Ga (3.5 mm) positrons have a larger average range in water than those of ^18^F (0.6 mm) (45).

Distant metastases of APMs occur in the liver, bone, lung, peritoneum and adrenal gland. The results of this study showed that ^68^Ga-FAPI was also better than ^18^F-FDG in the assessment of distant metastasis. Peritoneal metastases are common in APMs and can cause uncontrolled disease and even death (2). The uptake of ^68^Ga-FAPI-04 by peritoneal metastatic lesions is so avid that FAPI-04 clearly delineates and sensitively detects lesions (18). In a single-center retrospective study (18), the rate of positive detection of peritoneal metastases with ^68^Ga-FAPI was 93.2%, significantly higher than that with ^18^F-FDG (53.8%). Their results are similar to ours. In addition, the researchers reported that ^68^Ga-FAPI-04 PET/CT accurately detected advanced peritoneal lesions with a peritoneal cancer index ≥20 in 12 patients, all of whom were underestimated by ^18^F-FDG PET/CT (12/26 vs. 0/26, P < 0.001). It has been reported that a peritoneal cancer index score of 20 or more usually indicates a poor prognosis and the need for more aggressive treatment (46). This finding may be attributed to the invasion of peritoneal tissue by the tumor, which triggers a fibrotic response that leads to severe fibrosis (2). Thus, the advantage of FAPI in detecting peritoneal metastases of tumors may have a positive impact on patient management (47) and may be a promising tool for the assessment of peritoneal carcinomas (2, 18). FAPI PET/CT also shows strong potential for detecting liver, bone, and other metastases. The SUVmax and TBR of bone metastases in ^68^Ga-FAPI were significantly higher than those in ^18^F-FDG (p<0.001) (26). In the study of Fu et al. (18), ^68^Ga-FAPI and ^18^F-FDG showed similar abilities to detect bone metastases (108 vs. 104) and had complementary roles.

Heterogeneity across studies may be a potential source of bias in meta-analyses (48). The diversity of patient characteristics, methodological differences and overall quality of the study may all be sources of heterogeneity (48). Our results showed that ^68^Ga-FAPI had no heterogeneity in studies assessing primary tumor staging (I^2 =^ 22.58%, p=0.23), but ^18^F-FDG had heterogeneity (I^2 =^ 82.48%, p=0.00). Therefore, we performed a subgroup analysis of gastric and liver cancers in the primary tumor group and found improved heterogeneity of FAPI, while FDG remained, perhaps because more studies on FDG in tumors were not included. In the evaluation of non-primary tumors, the I^2^ value of the consistency test for all statistical indicators (SEN, SPE, DOR) was greater than 50%, so random effect models were used to combine effect sizes. Publication bias is a major concern in all meta-analyses, as studies reporting significantly positive results are more likely to be published than studies reporting negative results (49). In our meta-analysis, we used Deek funnel plots and Egger’s test to assess publication bias. Regardless of whether primary tumor staging or non-primary tumor metastases were detected, the funnel plots showed symmetry, indicating that there was no publication bias.

Our meta-analysis is innovative, and it is the first head-to-head comparison of the application of ^68^Ga-FAPI and ^18^F-FDG PET/CT to APMs. We evaluated not only the primary staging of ^68^Ga-FAPI and ^18^F-FDG PET/CT in the primary tumors but also the application of lymph nodes and distant metastases. We assessed the quality of the included studies using the QUADAS-2 tool; and no study was considered of low quality, and the overall quality of the studies was satisfactory. Certainly, our meta-analysis has some limitations. First, the number of published articles in the field was relatively small, which may be a source of bias. Second, heterogeneity among studies may affect the performance of pooled results. This may be because the subjects included in our study had tumors in different regions of the abdominal and pelvic cavities, with many types of diseases, but this was remedied after subgroup analysis. Third, there were many differences in the sample size and study design of the included studies, which may affect the reliability of the results. The high quality evidence provided by this meta-analysis may pave the way for opening the discussion on a change in the current diagnostic paradigm for solid gastrointestinal tumours. FAPI-imaging may be soon the standard of care in these tumours, given its advantages over FDG-imaging in this setting (50). However, generation of high-quality evidence is still warranted.

## 5 Conclusions

Our meta-analysis showed that ^68^Ga-FAPI and ^18^F-FDG PET/CT had a high overall diagnostic performance in detecting the primary staging and non-primary tumor metastasis of APMs, but the detection ability of ^68^Ga-FAPI was significantly higher than that of ^18^F-FDG. However, the ability of ^68^Ga-FAPI to diagnose lymph node metastasis is not very satisfactory, and is significantly lower than that of distant metastasis. In the future, ^68^Ga-FAPI will be a promising imaging model that may replace ^18^F-FDG for APM, but this still needs to be further confirmed by multicenter, large-sample, and prospective studies.

## Data availability statement

The original contributions presented in the study are included in the article/**Supplementary Material**. Further inquiries can be directed to the corresponding author.

## Author contributions

XL, HL and CG contributed to conception and design of the study. XL and HL organized the database. CG performed the statistical analysis. XL wrote the first draft of the manuscript. All authors contributed to manuscript revision, read, and approved the submitted version.
