# Supplementary material for: Comparison of 68Ga-FAPI and 18F-FDG PET/CT for the diagnosis of primary and metastatic lesions in abdominal and pelvic malignancies: A systematic review and meta-analysis
Source: Front Oncol. 2023 Feb 17;13:1093861. doi: 10.3389/fonc.2023.1093861 (PMC9982086; doi:10.3389/fonc.2023.1093861)
Supplement: Supplementary file 1 [file Table_1.docx]

**Supplementary Tables**

**Supplementary Table 1** The diagnostic efficacy of 68Ga-FAPI and 18F-FDG PET/CT in non-primary tumors (lymph nodes and distant metastases) of abdominal and pelvic malignancies.

|  | Non-primary tumors | | Lymph node metastasis | | Distant metastasis | |
| --- | --- | --- | --- | --- | --- | --- |
|  | ^68^Ga-FAPI | ^18^F-FDG | ^68^Ga-FAPI | ^18^F-FDG | ^68^Ga-FAPI | ^18^F-FDG |
| SEN | 0.717 (95% CI: 0.698-0.735), I^2^=99.1%, p=0.000 | 0.525 (95% CI: 0.505-0.546), I^2^=98.5%, p=0.000 | 0.421 (95% CI: 0.389-0.453), I^2^=99.4%, p=0.000 | 0.235 (95% CI: 0.207-0.264), I^2^=98.3%, p=0.000 | 0.918 (95% CI: 0.900-0.933), I^2^=98.2%, p=0.000 | 0.714 (95% CI: 0.686-0.741), I^2^=95.1%, p=0.000 |
| SPE | 0.891 (95% CI: 0.858-0.918), I^2^=83.0%, p=0.000 | 0.821 (95% CI: 0.786-0.853), I^2^=64.4%, p=0.002 | 0.908 (95% CI: 0.874-0.935),I^2^=82.6%, p=0.000 | 0.837 (95% CI: 0.799-0.870), I^2^=0.0%, p=0.573 | 0.844 (95% CI: 0.729-0.924), I^2^=52.6%, p=0.049 | 0.811 (95% CI: 0.691-0.900), I^2^=62.0%, p=0.015 |
| PLR | 4.351 (95% CI: 2.142-8.835), I^2^=74.8%, p=0.000 | 2.657 (95% CI: 1.639-4.309), I^2^=79.4%, p=0.000 | 5.447 (95% CI: 2.360-12.573), I^2^=65.6%, p=0.008 | 1.894 (95% CI: 0.758-4.728), I^2^=88.7%, p=0.000 | 2.223 (95% CI: 1.100-4.493), I^2^=21.8%, p=0.263 | 2.340 (95% CI: 1.484-3.690), I^2^=0.0%, p=0.462 |
| NLR | 0.121 (95% CI: 0.052-0.281), I^2^=98.0%, p=0.000 | 0.397 (95% CI: 0.215-0.734), I^2^=96.5%, p=0.000 | 0.123 (95% CI: 0.002-7.835), I^2^=99.7%, p=0.000 | 0.593 (95% CI: 0.301-1.169), I^2^=92.8%, p=0.000 | 0.040 (95% CI: 0.005-0.317), I^2^=85.6%, p=0.000 | 0.343 (95% CI: 0.178-0.660), I^2^=44.7%, p=0.093 |
| DOR | 60.435 (95% CI: 14.293-255.55), I^2^=84.5%, p=0.000 | 7.827 (95% CI: 3.178-19.277), I^2^=79.9%, p=0.000 | 35.860 (95% CI: 11.320-113.61), I^2^=44.7%, p=0.093 | 3.257 (95% CI: 0.656-16.176) I^2^=90.1%, p=0.000, | 72.059 (95% CI: 5.636-921.25), I^2^=73.1%, p=0.001 | 13.431 (95% CI: 5.759-31.322), I^2^=0.0%, p=0.495 |
| AUC | 0.946 | 0.841 | 0.934 | 0.877 | 0.850 | 0.777 |

SEN, sensitivity; SPE, specificity; PLR, positive likelihood ratio; NLR, negative likelihood ratio; DOR, diagnostic odds ratio, AUC, area under the curve.
